# Poor sleep quality is associated with exercise limitation in precapillary pulmonary hypertension

**DOI:** 10.1186/s12890-015-0005-3

**Published:** 2015-02-13

**Authors:** Henning Tiede, Janet Rorzyczka, Rio Dumitrascu, Michael Belly, Frank Reichenberger, Hossein Ardeschir Ghofrani, Werner Seeger, Jörg Heitmann, Richard Schulz

**Affiliations:** Department of Sleep Medicine, University of Giessen Lung Center, Klinikstr. 33, 35392 Giessen, Germany; Department of Pneumology, Asklepios Lung Center, Munich-Gauting, Germany

**Keywords:** Sleep, Pulmonary hypertension, Exercise capacity

## Abstract

**Background:**

Patients with precapillary pulmonary hypertension (PH) have been reported to suffer from poor sleep quality, however, if this is related to physical exercise performance has not yet been thoroughly investigated.

**Methods:**

Clinically stable out-patients with idiopathic pulmonary arterial hypertension (IPAH, n = 52) and chronic thromboembolic PH (CTEPH, n = 64) in NYHA classes II and III were prospectively enrolled. 54 healthy volunteers matched for anthropometric variables served as a control group. The Pittsburgh Sleep Quality Index (PSQI) was used to rate subjective sleep quality. In the PH patients, six-minute walk tests (6MWT) were performed to assess exercise capacity.

**Results:**

Poor sleep quality (i.e. a PSQI score > 5) occurred more frequently in PH (IPAH: n = 25 [48.1%], CTEPH: n = 39 [60.9%], controls: n = 10 [18.5%]; p < 0.01 when compared to controls). In addition, poor vs. good sleepers had significantly higher average NYHA class (IPAH: 2.6 ± 0.1 vs. 2.3 ± 0.1, CTEPH: 2.8 ± 0.1 vs. 2.3 ± 0.2; p < 0.01) and shorter 6MWT distances (IPAH: 338 ± 23 vs. 441 ± 29 m, CTEPH: 355 ± 15 vs. 413 ± 26 m; p < 0.05).

**Conclusions:**

Self-reported poor sleep quality is more common in PH than in healthy controls. Furthermore, it is related to reduced physical exercise capacity.

**Electronic supplementary material:**

The online version of this article (doi:10.1186/s12890-015-0005-3) contains supplementary material, which is available to authorized users.

## Background

It is well known that fatigue is a prominent clinical feature of precapillary pulmonary hypertension (PH, [1]). This may be considered to be due to right heart failure, however, it could also be the consequence of sleep disturbances occurring in these patients. In this context, significant proportions of PH patients have been found to suffer from night-time hypoxemia and sleep apnea [2-6]. Furthermore, mood disorders such as anxiety and depression which occur at higher frequencies in PH may lead to problems with falling asleep and/or maintaining normal sleep [7]. Finally, the restless legs syndrome has also been reported to be prevalent in PH [8].

In line with these assumptions, a recent study by Batal & colleagues reported poor sleep quality as assessed by the Pittsburgh Sleep Quality Index (PSQI) in 29 out of 40 patients with PH. Furthermore, correlations with depression, dyspnea and reduced quality of life were described [9]. However, apart from the relatively low number of patients, this study had some other limitations. First, the authors did not include a control group without PH. Second, the patients had a mix of etiologies leading to PH thus making it difficult to ascribe the findings to the presence of PH by itself.

Therefore, we aimed to evaluate sleep quality in a larger patient cohort with PH unbiased by any other underlying disorder and to compare it to a healthy control group matched for anthropometric variables. Furthermore, we looked for patient characteristics linked to the presence of poor sleep quality in PH. In particular, we wanted to test the hypothesis that those patients who are poor sleepers have more advanced disease and/or reduced physical exercise capacity.

For this purpose, we recruited patients with an ascertained diagnosis of idiopathic pulmonary arterial hypertension (IPAH) or chronic thromboembolic PH (CTEPH) as well as controls without PH and let them respond to standard sleep questionnaires.

## Methods

### Patient recruitment and assessment

The patients of the present study were investigated during an ambulatory visit to the pulmonary hypertension unit of the University of Giessen Lung Center, Germany between March and September 2012. The main inclusion criterion was the presence of PH as verified by right heart catheterisation performed within the preceding six months (mean pulmonary artery pressure [PAP] at rest of ≥ 25 mm Hg, pulmonary capillary wedge pressure [PCWP] < 15 mm Hg). Furthermore, the patients had to suffer from IPAH or CTEPH as determined by a diagnostic work-up including among others chest CT scans and ventilation/perfusion scintigraphy. Finally, they were requested to be clinically stable in New York Heart Association (NYHA) functional classes II or III. Exclusion criteria were the following : clinical signs of decompensated right heart failure (i.e. leg edema, syncope), known sleep-disorders such as sleep apnea, major neuropsychiatric diseases and intake of drugs interfering with central nervous system activity (for example benzodiazepines).

The anthropometric parameters of the patients were determined, i.e. age, sex and body mass index (BMI). All patients had pulmonary function tests with blood gas analysis from arterialized ear lobes.

The NYHA class was determined and six-minute walk tests (6MWT) were performed according to standard protocols [10]. Furthermore, serum brain natriuretic peptide (BNP) levels were measured. Finally, the intake of medications was evaluated, i.e. PH-specific therapy (phosphodiesterase-5-inhibitors, endothelin receptor antagonists, prostacyclin analogues), diuretics and oral anticoagulants and it was noted whether the patients were on long-term oxygen therapy (LTOT). Healthy volunteers matched with the PH patients for age, sex and BMI served as controls. They were recruited among the relatives and friends of one of the authors (R.D.). Based on their medical history, they were judged to be free of any chronic illness. In particular, PH was deemed to be absent (i.e. they did not suffer from symptoms suggestive of the disease such as exertional dyspnea, leg edema or syncope). The study protocol had been approved by the ethics committee of the medical faculty of the Justus-Liebig-University Giessen (approval number: 20/12) and all patients and controls had given their informed written consent.

### Sleep questionnaires

The patients were asked to fill in self-administered questionnaires of sleep quality and daytime sleepiness. Sleep quality was evaluated by the PSQI which has a time window of 4 weeks. It consists of 7 domains with a total of 19 questions. In each domain a score of 0-3 can be achieved. The scores of all domains are summarized (range: 0-21) with higher values indicating poorer sleep quality ([11]; Additional file 1). Based on prior validations of the PSQI in psychiatric populations, a value of > 5 is regarded as evidence for poor sleep quality. Apart from sleep quality, subjective sleep duration was also extracted from the answers of the PSQI.

The Epworth Sleepiness Scale (ESS) was used to determine the level of daytime sleepiness. In this questionnaire, the probability of falling asleep in different situations (n = 8) has to be rated on a 4-point scale (0 = ‘never’ to 3 = ‘high probability’). The maximal score of the ESS is 24 and values of > 10 are considered to indicate excessive daytime sleepiness ([12]; Additional file 2). The questions of the ESS relate to the “last time” without any further specifications. All patients and controls who were approached to take part in the study consented to complete the questionnaires. The results were analyzed by an investigator blinded to the status of the study participants (J.R.).

### Data analysis

Data are presented as n/% or mean ± standard error of the mean as appropriate. ANOVA with Tukey post-hoc test, student’s *t*-test, and Chi square test were used for testing of statistical significance of differences between the groups in normally distributed parameters. Mann Whitney *U* test was performed as non-parametric test. SPSS 19.0 was used for calculating statistics. A p-value of < 0.05 was regarded as statistically significant.

## Results

### Characteristics of patients and controls

The patient characteristics are summarized in Table 1. A total of 52 IPAH patients and 64 CTEPH patients were enrolled.

**Table 1:** **Patient characteristics**

	**IPAH**	**CTEPH**
Number of patients	52	64
Age (yrs.)	58.2 ± 2.0	62.7 ± 1.7
Females (n/%)	39/75.0	39/60.9
BMI (kg/m^2^)	26.1 ± 0.9	27.4 ± 0.7
NYHA FC	2.6 ± 0.1	2.6 ± 0.1
BNP (pg/ml)	171 ± 34	120 ± 29
6MWT distance (m)	393 ± 20	375 ± 14
Mean PAP (mm Hg)	45.3 ± 1.9	42.0 ± 1.5
PVR (dyn)	718 ± 62	647 ± 41
CI (l/min/m^2^)	2.7 ± 0.1	2.3 ± 0.1
CVP (mm Hg)	6.2 ± 0.6	6.2 ± 0.5
PCWP (mm Hg)	8.5 ± 0.5	8.9 ± 0.4
FEV_1_ (% of pred.)	81.9 ± 3.0	79.0 ± 2.3
IVC (% of pred.)	92.5 ± 2.9	87.7 ± 1.9
FEV_1_/IVC (% of pred.)	91.4 ± 1.6	92.1 ± 1.6
DLCO (% of pred.)	70.5 ± 2.1	74.6 ± 1.9
pO_2_ (mm Hg)	68.2 ± 1.6	65.9 ± 1.1
pCO_2_ (mm Hg)	34.3 ± 0.8	35.7 ± 0.8
LTOT (n / %)	20/38.5	26/40.6
Oral anticoagulation (n/%)	37/71.2	63/98.4
Diuretics (n/%)	43/82.7	47/73.4
No vasodilator therapy (n/%)	11/21.2	16/25.0
Vasodilator monotherapy (n/%)	24/46.2	33/51.6
Vasodilator combination therapy (n/%)	17/32.7	15/23.4

*Abbreviations:*

BMI: body mass index, BNP : brain natriuretic peptide, CI: cardiac index, CTEPH: chronic thromboembolic pulmonary hypertension, CVP: central venous pressure, DLCO: diffusion capacity for carbon monoxide, FEV_1_: forced exspiratory volume in one second, IPAH: idiopathic pulmonary arterial hypertension, IVC: inspiratory vital capacity, LTOT: long-term oxygen therapy, NYHA FC: New York Heart Association functional class, PAP: pulmonary artery pressure, PCWP: pulmonary capillary wedge pressure, pO_2_ / pCO_2_: partial pressure of oxygen and carbon dioxide, PVR: pulmonary vascular resistance, 6MWT: six-minute walk test.

In both groups anthropometric parameters were similar with a mean age around 60 years, a preponderance of females and a BMI lying in the upper normal range. Based on the inclusion criteria of the study, all patients were in NYHA classes II or III. On an average, they had only mildly elevated serum BNP levels and relatively well preserved physical activity (i.e. the average 6MWT distance was above 350 m). Right heart catheterization had shown quite severe PH with an average mean PAP > 40 mm Hg in both groups. Except for a moderate impairment of diffusion capacity pulmonary function was normal. Blood gas analysis yielded mild hypoxemia with preserved oxygen saturation (i.e. SaO_2_ above 90%) and pCO_2_ levels in the lower normal range (i.e. on an average, they were about 35 mm Hg in both groups of PH patients). About 40% of the IPAH and CTEPH patients were under LTOT. The majority of them took oral anticoagulants and diuretics and were already treated by pulmonary vasodilator drugs at the time of study inclusion (either as monotherapy or combination therapy).

The healthy control group consisted of 54 subjects. They were matched with the PH patients with regard to age, gender and BMI (age: 58.9 ± 1.8 yrs, n = 34 females [63.0%], BMI: 26.4 ± 0.8 kg/m^2^, p = n.s. in comparison to the IPAH and CTEPH groups).

### Sleep characteristics of PH patients vs. controls

The sleep characteristics of PH patients vs. controls are shown in Table 2. The average sleep duration was similar in both groups with approximately 7 hours per night. Overall, sleep duration was highly variable with significant proportions of patients and controls reporting short or long sleep duration (i.e. ≤ 6 and ≥ 8 hours per night, data not shown).

**Table 2:** **Sleep characteristics in PH patients vs. controls**

	**IPAH (n=52)**	**CTEPH (n=64)**	**Controls (n=54)**
sleep duration (h)	7.1 ± 0.2	7.0 ± 0.2	6.9 ± 0.2
PSQI score	6.1 ± 0.5^§^	6.8 ± 0.5^§^	4.0 ± 0.4
PSQI score > 5 (n / %)	25/48.1^§^	39/60.9^§^	10/18.5
ESS score	6.8 ± 0.7	5.7 ± 0.5	5.2 ± 0.4
ESS score > 10 (n / %)	14/26.9^§/^^#^	6/9.5	4/7.

*Abbreviations:*

ESS: Epworth Sleepiness Scale, PSQI: Pittsburgh Sleep Quality Index, for other abbreviations see Table 1.

^§^p < 0.01 in comparison to the control group.

^#^p < 0.01 in comparison to the CTEPH group.

The mean PSQI score was significantly higher in the PH groups than in the controls. Furthermore, poor sleep quality (as defined by a PSQI score > 5) was more often observed in PH. 64 of the 116 patients with PH (i.e. 55.2%) suffered from impaired sleep quality. The average score of the ESS also tended to be higher in patients than in controls but these differences did not reach statistical significance. Daytime sleepiness (i.e. an ESS score > 10) was observed in 14 out of 52 patients (i.e. 26.9%) with IPAH but in only a few CTEPH patients and control subjects.

### Relation of sleep variables to characteristics of PH patients

**Figure 1:**
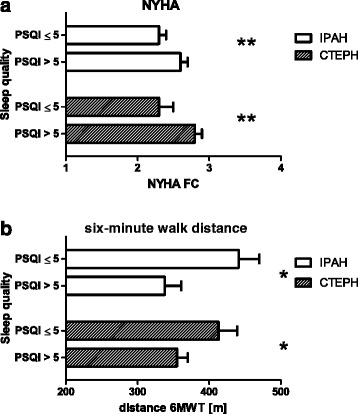
**Relation of sleep quality to exercise performance (a) NYHA functional class and (b) six-minute walk distance.** For abbreviations see Tables 1 and 2. *p < 0.05 in comparison to patients with a PSQI score ≤ 5. **p < 0.01 in comparison to patients with a PSQI score ≤ 5.

The PH patients with vs. without poor sleep quality did neither differ with regard to anthropometric variables nor the severity of the disease as reflected by serum BNP levels and hemodynamics. The same was true for the use of LTOT and medications (data not shown). However, those patients who were judged to have impaired sleep quality were characterized by significantly higher average NYHA class (IPAH: 2.6 ± 0.1 vs. 2.3 ± 0.1, CTEPH: 2.8 ± 0.1 vs. 2.3 ± 0.2; p < 0.01 respectively; Figure 1a). In addition, poor sleepers had significantly shorter 6MWT distances (IPAH: 338 ± 23 vs. 441 ± 29 m, CTEPH: 355 ± 15 vs. 413 ± 26 m; p < 0.05 respectively; Figure 1b). The PH patients with short/long vs. normal sleep duration and those with vs. without excessive daytime sleepiness did not differ with regard to any of the characteristics investigated (data not shown).

## Discussion

The main findings of the present study were that more than half of the patients with PH suffered from poor sleep quality and that poor sleepers were characterized by higher average NYHA class and shorter 6MWT distances.

Poor sleep quality was frequently encountered in our PH patients which is in line with a preceding study by Batal & colleagues [9]. However, in contrast to this study, we employed more stringent recruitment criteria (i.e. only IPAH and CTEPH patients) and included a carefully selected control group of age-, sex- and BMI-matched healthy volunteers. It is also noteworthy that we only investigated clinically stable out-patients in NYHA classes II and III. We presume that poor sleep quality would have been even more frequent if we had also enrolled patients who were hospitalized and/or in NYHA class IV.

An interesting and novel observation was that poor sleep quality was associated with significantly higher average NYHA class and shorter 6MWT distances. This is in contrast to the results of Batal et al. [9] which may be due to the smaller sample size of this earlier study. Both NYHA class and 6MWT distance are important determinants of survival in patients with PH [13-15]. Given the findings of the present study, poor sleep quality may constitute an adverse prognostic sign in PH just as it has recently been recognized in patients with chronic left heart failure [16].

The 6MWT has been widely used as an end point in drug trials of PH with relatively modest changes regarded to be clinically significant [17]. The fact that poor sleepers reached 6MWT distances up to 100 meters shorter than those of good sleepers clearly suggests that sleep quality is an important confounder when conducting studies of PH-specific therapy.

Apart from sleep quality we also evaluated daytime sleepiness and sleep duration in our PH patients. As already stated, fatigue is one of the leading symptoms of PH [1]. It should be realized, however, that fatigue and sleepiness describe two different clinical situations. Fatigue means a feeling of exhaustion leading to limitations of bodily functions. In contrast, sleepiness is characterized by reduced alertness with an uncontrollable propensity to fall asleep. In the study by Batal et al. daytime sleepiness was observed in 26% of the patients investigated [9]. At least in the IPAH group we found a quite similar percentage of patients considered to be sleepy.

The average sleep duration was about 7 hours per night in the PH patients and the controls. Studies performed in the community have found that individual sleep duration shows a widespread normal distribution with a peak occurring at 6-8 hours per night [18]. Therefore, the sleep durations observed in our study can be considered to lie in the normal range. In contrast to poor sleep quality, neither daytime sleepiness nor short/long sleep duration were associated with reduced physical exercise capacity or any other patient characteristics.

Our study has some possible limitations. First, we did not perform polysomnography and therefore could not gain insight into the patients’ sleep architecture, i.e. objective sleep quality. Second, we did not carry out multiple sleep latency tests which would have enabled us to rate the degree of daytime sleepiness of our patients. Third, it should be realized that the PSQI and ESS have not yet been validated in PH. Nevertheless, we employed the cut-off values which have been reported in other patient populations to separate individuals with vs. without poor sleep quality/daytime sleepiness to describe our findings. Fourth, on the basis of our data, it is not possible to exactly identify the causes of poor sleep quality in PH.

At least, it does not seem to be related to demographic variables or the degree of hemodynamic compromise as these characteristics did not differ between patients with vs. without impaired sleep quality. One major determinant may be the presence of depression and/or reduced quality of life as suggested by the data of Batal et al. [9]. Likewise, sleep-related breathing disorders which have been reported to occur in a considerable proportion of PH patients may contribute to poor sleep quality [2-6]. We suggest that these issues should be addressed by future studies.

A final drawback of the present study may be that we chose healthy volunteers as a control group. One may argue that any chronic illness can cause poor sleep quality and that our findings are nonspecific for PH. Indeed, this seems to be the case as for example suggested by our own observations in a group of adults with cystic fibrosis [19]. However, the aim of our study was to investigate if PH patients have poor sleep quality in comparison to what is judged to be “normal” and therefore we included people free from any chronic disease as controls.

## Conclusion

In conclusion, self-reported poor sleep quality is more common in clinically stable out-patients with PH than in healthy controls matched for anthropometric variables. Furthermore, it is related to exercise limitation as reflected by higher average NYHA class and shorter 6MWT distances. Although more studies are needed in this area of research, the present findings suggest that more attention should be paid to sleep-related symptoms in patients with PH.

## Additional files

Additional file 1:
**Pittsburgh sleep quality index.**
Additional file 2:
**Epworth sleepiness scale.**
