# Supplementary material for: Poor sleep quality is associated with exercise limitation in precapillary pulmonary hypertension
Source: BMC Pulm Med. 2015 Feb 13;15:11. doi: 10.1186/s12890-015-0005-3 (PMC4342878; doi:10.1186/s12890-015-0005-3)
Supplement: Additional file 1: — Pittsburgh sleep quality index. [file 12890_2015_5_MOESM1_ESM.pdf]

Subject's Initials________________ID#__________________Date_______________Time_____AM / PM

PITTSBURGH SLEEP QUALITY INDEX

INSTRUCTIONS:
The following questions relate to your usual sleep habits during the past month only. Your answers should indicate the most accurate reply for the majority of days and nights in the past month. Please answer all questions.

1. During the past month, what time have you usually gone to bed at night?

   BED TIME ___________

2. During the past month, how long (in minutes) has it usually taken you to fall asleep each night?

   NUMBER OF MINUTES ___________

3. During the past month, what time have you usually gotten up in the morning?

   GETTING UP TIME ___________

4. During the past month, how many hours of actual sleep did you get at night? (This may be different than the number of hours you spent in bed.)

   HOURS OF SLEEP PER NIGHT ___________

For each of the remaining questions, check the one best response. Please answer all questions.

5. During the past month, how often have you had trouble sleeping because you . . .

a) Cannot get to sleep within 30 minutes

Not during the past month_____	Less than once a week_____	Once or twice a week_____	Three or more times a week_____

b) Wake up in the middle of the night or early morning

Not during the past month_____	Less than once a week_____	Once or twice a week_____	Three or more times a week_____

c) Have to get up to use the bathroom

Not during the past month_____	Less than once a week_____	Once or twice a week_____	Three or more times a week_____

d) Cannot breathe comfortably

Not during the past month_____	Less than once a week_____	Once or twice a week_____	Three or more times a week_____

e) Cough or snore loudly

Not during the past month_____	Less than once a week_____	Once or twice a week_____	Three or more times a week_____

f) Feel too cold

Not during the past month_____	Less than once a week_____	Once or twice a week_____	Three or more times a week_____

g) Feel too hot

Not during the past month_____	Less than once a week_____	Once or twice a week_____	Three or more times a week_____

h) Had bad dreams

Not during the past month_____	Less than once a week_____	Once or twice a week_____	Three or more times a week_____

i) Have pain

Not during the past month_____	Less than once a week_____	Once or twice a week_____	Three or more times a week_____

j) Other reason(s), please describe____

____

How often during the past month have you had trouble sleeping because of this?

Not during the past month_____	Less than once a week_____	Once or twice a week_____	Three or more times a week_____

6. During the past month, how would you rate your sleep quality overall?

Very good ____________

Fairly good ____________

Fairly bad ____________

Very bad ____________

7. During the past month, how often have you taken medicine to help you sleep (prescribed or "over the counter")?

Not during the past month_____	Less than once a week_____	Once or twice a week_____	Three or more times a week_____

8. During the past month, how often have you had trouble staying awake while driving, eating meals, or engaging in social activity?

Not during the past month_____	Less than once a week_____	Once or twice a week_____	Three or more times a week_____

9. During the past month, how much of a problem has it been for you to keep up enough enthusiasm to get things done?

No problem at all	__________
Only a very slight problem	__________
Somewhat of a problem	__________
A very big problem	__________

10. Do you have a bed partner or room mate?

No bed partner or room mate	__________
Partner/room mate in other room	__________
Partner in same room, but not same bed	__________
Partner in same bed	__________

If you have a room mate or bed partner, ask him/her how often in the past month you have had . . .

a) Loud snoring

Not during the past month_____	Less than once a week_____	Once or twice a week_____	Three or more times a week_____

b) Long pauses between breaths while asleep

Not during the past month_____	Less than once a week_____	Once or twice a week_____	Three or more times a week_____

c) Legs twitching or jerking while you sleep

Not during the past month_____	Less than once a week_____	Once or twice a week_____	Three or more times a week_____

d) Episodes of disorientation or confusion during sleep

Not during the past month_____ Less than once a week_____ Once or twice a week_____ Three or more times a week_____

e) Other restlessness while you sleep; please describe________________________________

____

Not during the past month_____ Less than once a week_____ Once or twice a week_____ Three or more times a week_____

© 1989, University of Pittsburgh. All rights reserved. Developed by Buysse,D.J., Reynolds,C.F., Monk,T.H., Berman,S.R., and Kupfer,D.J. of the University of Pittsburgh using National Institute of Mental Health Funding.

Buysse DJ, Reynolds CF, Monk TH, Berman SR, Kupfer DJ: Psychiatry Research, 28:193-213, 1989.
