# Supplementary figures and images for: Poor sleep quality is associated with exercise limitation in precapillary pulmonary hypertension
Source: BMC Pulm Med. 2015 Feb 13;15:11. doi: 10.1186/s12890-015-0005-3 (PMC4342878; doi:10.1186/s12890-015-0005-3)

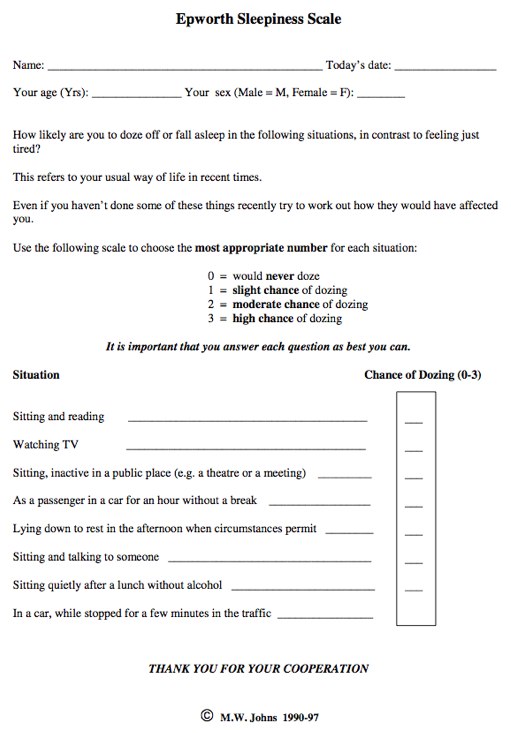

Supplement: Additional file 2: — Epworth sleepiness scale. [file 12890_2015_5_MOESM2_ESM.doc]
